# A Giant Primary Retroperitoneal Serous Cystadenoma: Case Report and Review of Retroperitoneal Cysts

**DOI:** 10.1055/s-0037-1599820

**Published:** 2017-03-16

**Authors:** Amit Mori, Kinesh Changela, Dhuha Alhankawi, Alexander Itskovich, Ahmar Butt, Madhavi Reddy

**Affiliations:** 1 Division of Gastroenterology and Hepatology, The Brooklyn Hospital Center, Clinical Affiliate of Mount Sinai Hospital, Brooklyn, New York; 2 Division of Internal Medicine, The Brooklyn Hospital Center, Clinical Affiliate of Mount Sinai Hospital, Brooklyn, New York; 3 Division of Surgery, The Brooklyn Hospital Center, Clinical Affiliate of Mount Sinai Hospital, Brooklyn, New York

**Keywords:** primary, retroperitoneal, serous, cystadenomas, cystic

## Abstract

Primary retroperitoneal serous cystadenomas (PRSCs) are rare cystic lesions whose pathogenesis is currently not well understood. Although the vast majority of tumors are benign, early recognition and resection is necessary to avoid malignant transformation, rupture, and secondary infection. Here we present the case of a 79-year-old woman who presented with confusion, visual hallucinations, and a history of fall. As part of the work-up for abdominal distension, computed tomography scan of the abdomen and pelvis was performed, which revealed a right-sided retroperitoneal cystic lesion measuring 26.6 × 16.7 cm in size. The lesion was resected laparoscopically, and the surgical specimen measured 28 × 17 cm. Histology revealed a serous cystadenoma. The postsurgical course was uneventful, and no radiological recurrence was noted on 3 months follow-up. Very few primary retroperitoneal cystic lesions have been reported in the literature. Most lesions are benign and predominantly occur in females. They may remain asymptomatic for long periods of time and are usually discovered when they reach very large in size. In rare cases, these lesions may have malignant potential. Diagnosis of PRSC should be considered in the differential diagnosis of all retroperitoneal cysts.

Primary retroperitoneal serous cystadenoma (PRSC) is an extremely uncommon lesion of retroperitoneum. Very few case reports of this entity have been described in the literature. The pathogenesis is not well understood, although one of the proposed hypotheses considers it to be an embryological remnant of the urogenital apparatus with epithelial and mesothelial tissues. 1 These cysts oftentimes attain very large size before becoming symptomatic. Our case review is focused on the diagnostic, therapeutic, and pathological findings of this rare entity.

## Case Presentation

A 79-year-old female presented to the emergency room with confusion, agitation, and visual hallucinations after sustaining a fall. Although the patient was a poor historian, her family members did not report prior history of abdominal complaints including trauma, fever, weight loss, abdominal mass, or surgery. Abdominal examination revealed soft, nontender, palpable fullness in the paraumbilical region. Gastroenterology service was consulted for a nontender abdominal fullness on physical examination as well as laboratory findings of severe iron deficiency anemia. The rest of her laboratory findings were unremarkable.

**Fig. 1:**
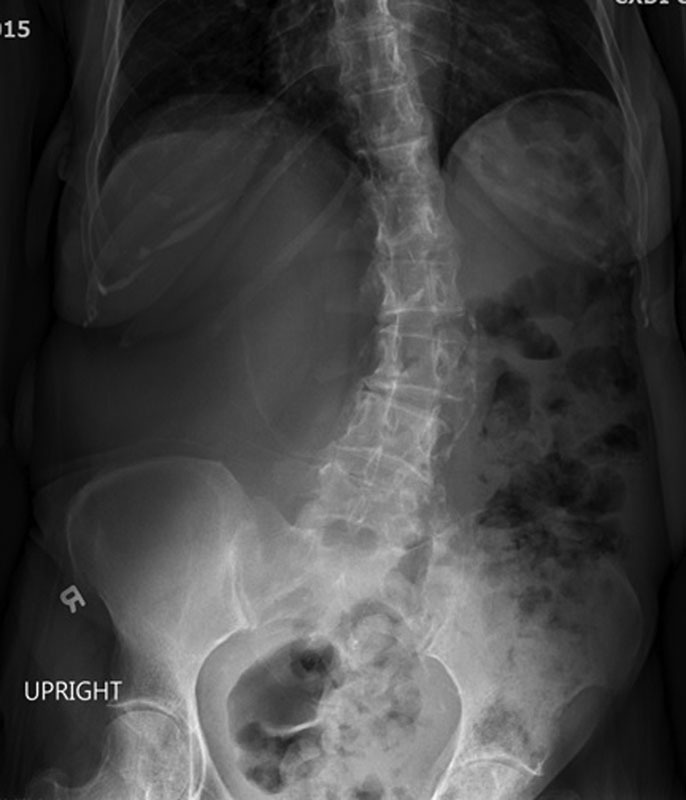
Plain upright abdominal film showing displaced bowel loops to the left with large amount of stool in the colon.

**Fig. 2:**
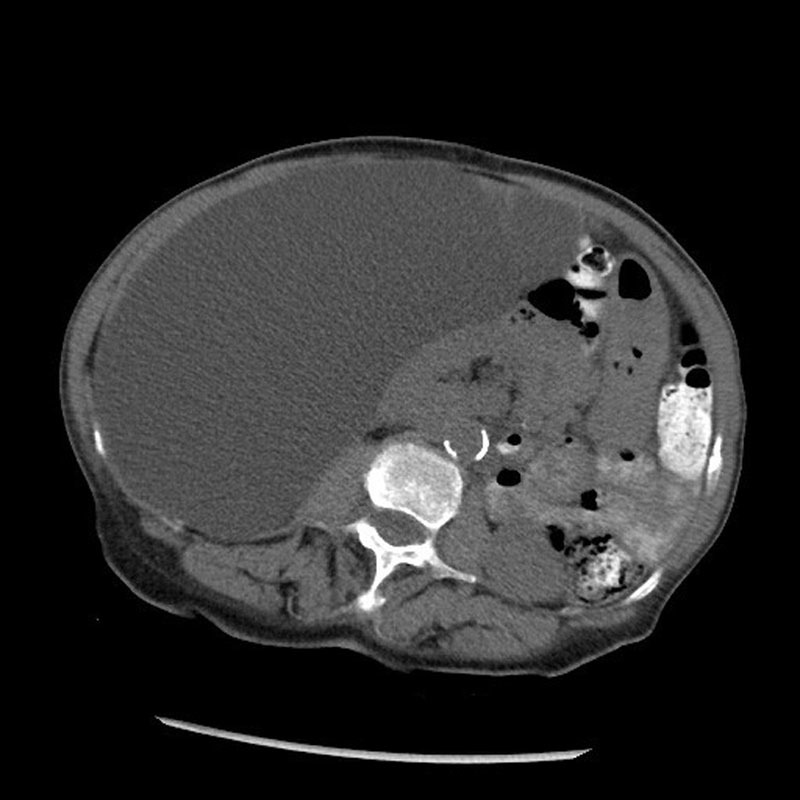
Abdominal computed tomography shows a large right retroperitoneal cystic lesion with displacement of the right kidney and bowel loops to the left.

**Fig. 3:**
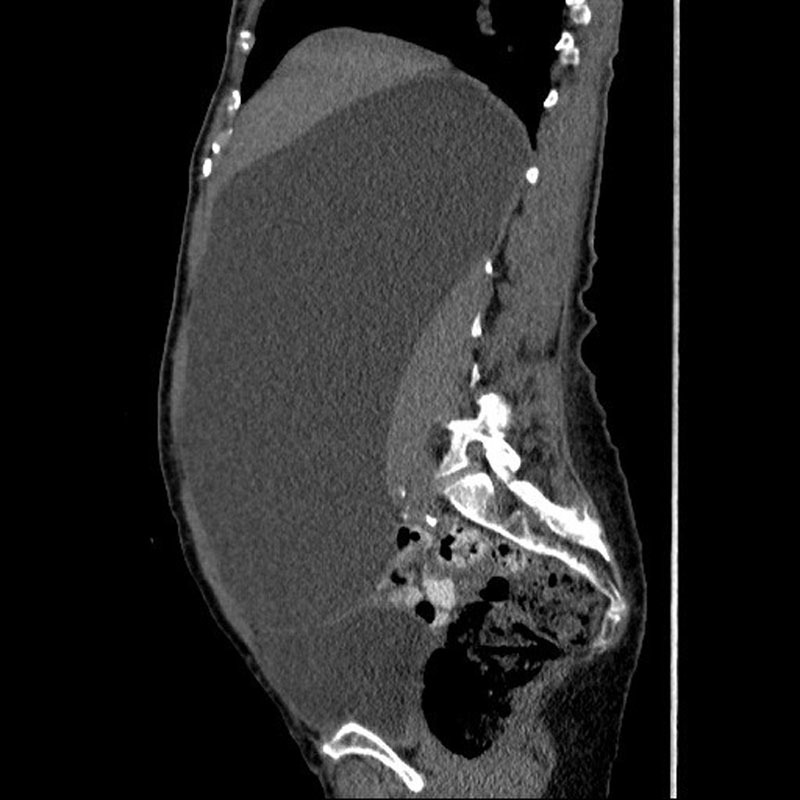
Abdominal computed tomography (sagittal plane) showing a giant retroperitoneal cyst.

A supine and upright abdominal film showed bowel loops displaced to the left with a large amount of stool in the colon ( Fig. 1 ). A noncontrast computed tomography (CT) of the abdomen and pelvis revealed a large right retroperitoneal cystic structure measuring at least 26.6 × 11.7 × 16.7 cm. The cyst was displacing the right kidney and right colon medially ( Figs. 2 and 3 ). These findings were consistent with a giant retroperitoneal cyst (RPC). Though her presenting complaints were attributed to her history of fall with possible concussion, the diagnosis of RPC was found via thorough physical exam revealing a large, palpable abdominal mass.

**Fig. 4:**
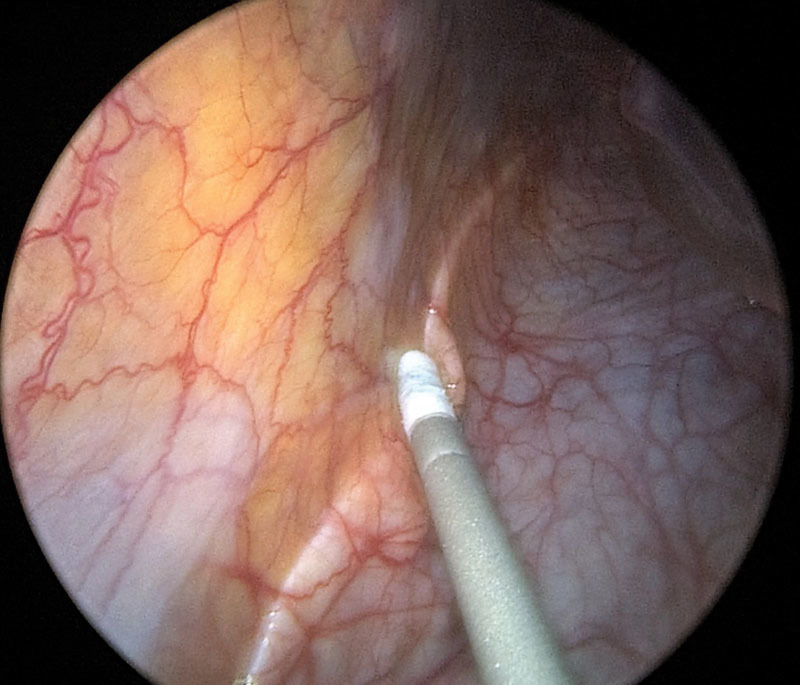
Laparoscopic view of the cyst wall noted behind the posterior peritoneum.

**Fig. 5:**
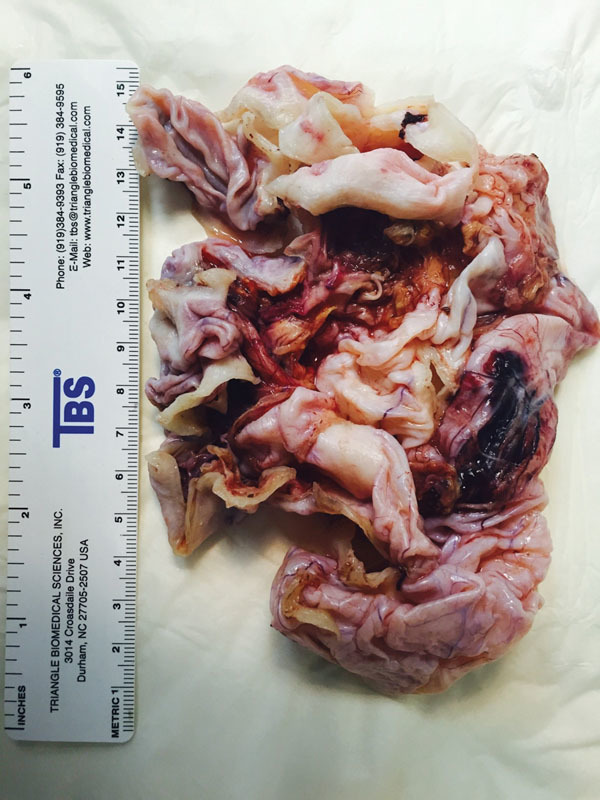
Macroscopic view of the surgical specimen shows thick cystic wall.

**Fig. 6:**
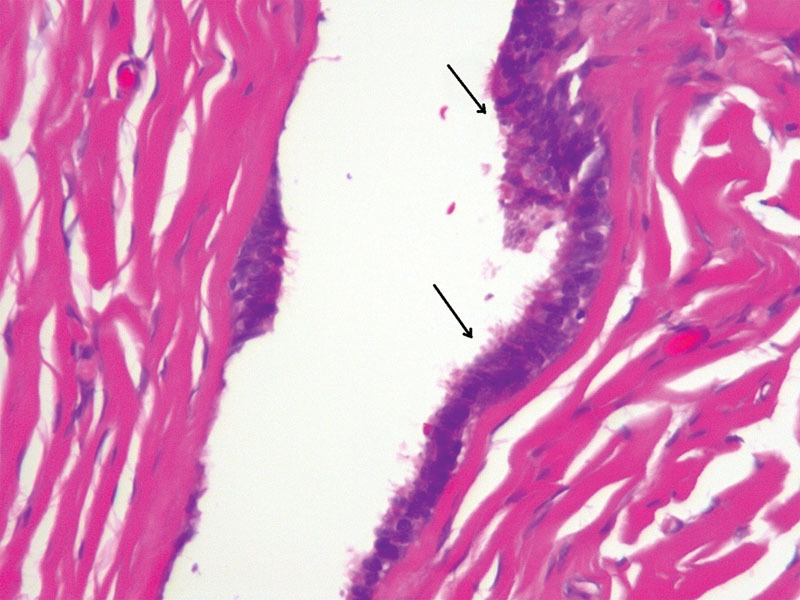
Serous cystadenoma: fibrous tissue lined by mostly single-layer cuboidal or columnar serous epithelium (arrows) with tubal metaplasia. There is no ovarian tissue or mucinous epithelium. No cellular atypia (H & E x 100).

The patient was taken to the operating room with a high suspicion that the CT findings were consistent with a giant liver cyst. Upon entering the abdomen using a Hasson technique and placing a 12-mm trocar, a diagnostic laparoscopy revealed a large cyst with a blue hue. Given that the CT findings showed no evidence of nodularity, septation, or intracystic masses and the laparoscopic findings were consistent, the suspicion of malignancy was very low. Upon drainage of the cyst, it was clearly noted that it was easily separable from the posterior liver and was in fact retroperitoneal in origin. The posterior peritoneum was incised. The wall of the cyst was carefully dissected from the anterolateral wall of the kidney, adrenal, and the posterior wall of the ascending colon keeping in mind the significant distortions in anatomy secondary to its size. A plane between the cyst and the posterior peritoneum was established, and the cyst was separated using a combination of electrocautery and sharp dissection. The cyst was then placed in an endocatch bag and removed through the 12-mm port. Thus, the patient underwent a complete cyst excision using a laparoscopic transperitoneal approach. Intraoperatively, the retroperitoneal location of the cyst was confirmed as the cyst wall was separated from visceral peritoneum with ease ( Fig. 4 ). Macroscopically, the cyst appeared to have thick walls and measured approximately 28 × 17 cm in size and containing 6 L of clear liquid ( Fig. 5 ). Histopathology revealed a benign serous cystadenoma ( Fig. 6 ). Should there have been a preoperative suspicion of malignancy based on imaging or, diagnostic laparoscopy, an open approach with en bloc resection would have been undertaken. A subsequent CT revealed interval removal of large RPC with properly repositioned loops of bowel. The patient followed up in 3 months with no further surgical complications or recurrence. We believe that the patient’s presenting symptoms were attributable to her fall, concussion, and dehydration that resolved with hydration and supportive therapy and not related to the findings of RPC or its surgical removal.

## Discussion

The retroperitoneal space is bounded anteriorly by the posterior part of parietal peritoneum, posteriorly by the psoas and quadratus lumborum muscles, as well as the spine, superiorly by the diaphragm, and inferiorly by the muscular wall of the pelvis. 1 The large size of this space enables lesions to grow and remain asymptomatic for a long period of time. Only those cysts derived from this space without direct contact with other anatomical organs are included in the category of RPCs and are discussed here. Retroperitoneal cystadenoma was first described by Staehlin in 1915. 2 Although primary retroperitoneal mucinous cystadenoma is more common, to our knowledge, there are only a few cases of PRSC that have been described in the literature. 3 4 5 6 7 8 Sharatz et al reported the largest case of benign serous type cystadenoma measuring 18.7 × 15.4 × 10 cm 5 ; our case is the largest reported PRSC in the literature.

Primary retroperitoneal cystic lesions are extremely rare because of a lack of epithelial cells in this region. Their incidence is difficult to estimate. The pathogenesis of primary RPCs is not well understood. Several hypotheses are described in the literature, such as: (1) coelomic epithelial metaplasia, 9 (2) remnants of embryonal urogenital apparatus (pronephric, mesonephric, metanephric, and Mullerian, depending on their cell line of origin), 10 (3) heterotopic ovarian tissue, 7 (4) germ cell layers (cystic teratomas), 11 and (5) enteric duplication cyst. 12 Based on their etiology RPCs can be further divided into traumatic (urinoma, hematoma), parasitic, lymphatic, and cystic changes of a solid neoplasm (e.g., neurilemmoma, paraganglioma, sarcoma). Based on their malignant potential, RPCs can be neoplastic and nonneoplastic ( Table 1 ). 13 14 Our literature review also reveals that based on their clinical course and microscopic appearance, primary retroperitoneal epithelial lesions can be further classified into benign (serous and mucinous cystadenoma), lesions with borderline malignancy, 3 and malignant (serous and mucinous cystadenocarcinoma). 15

**Table 1:** Classification and characteristics of retroperitoneal cystic lesions

Type of lesion	Gender	Imaging appearance	Demographic features
Serous/mucinous cystadenoma a	Female	Homogeneous, unilocular, thin-walled cystic mass	Symptoms based on size, very low risk of recurrence with complete cyst excision, rarely elevated CA125 and CA19-9 levels in the clear fluid 15 16
Mullerian cyst a	Female	Unilocular or multilocular, thin walled with clear fluid	Obese patients, history of irregular menses, microscopically cyst wall has thick smooth muscle and columnar epithelial cells
Cystic teratoma a	Both genders with little female predominance	Hypoattenuating fat within the cyst with sometimes typical wall calcifications	Mixed germline tissue on microscopy, young age, low malignant potential
Cystic lymphangioma a	Male	Large, elongated, multilocular, thin-walled, complex cystic mass, may cross into adjacent compartment	Clear or milky fluid, single layer of endothelial cells with lymphoid aggregates
Cystic mesothelioma a	Female	Unilocular or multilocular thin- walled cyst	Not related to prior asbestos exposure, thin-walled cysts with watery fluid on pathology, potential for local recurrence but no metastases
Tailgut cyst a	Female	Well-defined, multicystic mass with wide range of attenuation, thick walled if infected, may compress rectum, rare thin calcifications	Embryonic hindgut in origin, occurs between rectum and sacrum. Microscopically, cyst wall may show several different types of epithelium. Middle-aged women may be complicated by infection and/or malignant transformation
Omental/mesenteric cyst a	Both genders	Thin or thick-walled, uni- or multilocular, anywhere from duodenum to the rectum	Bimodal age distribution (pediatrics and middle aged), small bowel mesentery origin more common 20
Epidermoid cyst a	Female	Thin-walled, unilocular with fluid attenuation, presacral retroperitoneal location	Ectodermal in origin, may occur anywhere, middle-aged women, may present with local mass effect (e.g., pain, palpable mass). Microscopically has stratified squamous epithelium with mixture of water, keratin, skin debris, cholesterol
Paraganglioma a	Slight female predominance	Homogenous, soft-tissue attenuation or central areas of low attenuation, rarely with internal hemorrhage subsequently forming thick capsule mimicking cystic lesion	Arise from neural crest cells and sympathetic chain, may produce catecholamines and lead to hypertension, middle-aged patient, autosomal dominant and may be associated with MEN syndrome
Neurilemmoma a	Female	Thick-walled, located in paravertebral space or pelvic retroperitoneum	Encapsulated tumor from peripheral nerve sheaths (Schwann cells), 20–50 y of age, may be associated with neurofibromatosis type-1
Urinoma b	Both genders	CT and MRI show water attenuated fluid collection, hypointense T1-weighted and hyperintense T2-weighted images on MRI, IVP shows contrast extravasation into retroperitoneal tissues 16	History of blunt trauma, usually located in perirenal space, usually has associated hydronephrosis, percutaneous drainage is diagnostic and therapeutic
Hematoma b	Both genders	Unenhanced CT shows abnormal soft tissue density that may compress adjacent structures, spiral CT better in assessing acute active bleed as it shows a jet of contrast extravasation 23	History of trauma, coagulopathy, ruptured aortic aneurysm. Conservative management in small, stable hematomas. Surgical management for large, unstable hematomas 23
Pancreatic pseudocyst b	Both genders	Well-circumscribed, usually round or oval peripancreatic fluid collections of homogeneously low attenuation that are usually surrounded by a well-defined enhancing wall 24	Clinical history of pancreatitis, abdominal pain or palpable mass, elevated amylase and lipase levels in blood test Large symptomatic cysts require endoscopic, percutaneous or surgical drainage.
Nonpancreatic pseudocyst b	Both genders	Unilocular or multilocular fluid-filled complex cystic lesions with thick walls 18	Rare lesions arising from mesentery and omentum. May contain serous or purulent fluid with or without blood. Microscopically, cyst wall lacks cell lining and consists of connective tissue with chronic inflammatory changes 18 25
Lymphocele b	Both genders	Unilocular or multilocular fluid-filled complex cystic lesions with thick walls 18	Occurs in up to 30% of patients after lymphadenectomy and in 18% of patients after renal transplantation. Symptoms mostly due to mass effect of adjacent structures or secondary infection 25

Abbreviations: CT, computed tomography; IVP, intravenous pyelogram; MEN, multiple endocrine neoplasia; MRI, magnetic resonance imaging.

a Neoplastic.

b Nonneoplastic.

Clinical presentation of retroperitoneal cystic lesions may vary depending on their location and size. Most commonly, cysts present as a palpable mass or abdominal pain. Constitutional symptoms such as fever, changes in appetite, and weight loss may be present especially in malignant lesions. Our patient had a palpable mass on examination, leading to the further work-up and diagnosis.

There are no pathognomonic signs, symptoms, and laboratory or imaging findings reported in the literature to confirm the diagnosis. However, few case reports suggest association with elevated blood levels of carcinoembryonic antigen (CEA), fetoprotein, CA125, CA19-9, and CA15-3. 6 8 15 16 17 There are also case reports that may suggest some diagnostic value of the presence of CA125, CA19-9, 8 and CEA 15 in the aspirated cystic fluid. Certain imaging features that may suggest retroperitoneal location include location of the lesion posterior to the psoas muscle, anterior displacement of the rectum as well as anterior or medial displacement of major iliac vessels, ureter, and the iliopsoas muscle. It is suggested that magnetic resonance imaging (MRI) is a better modality than CT in identifying retroperitoneal lesions as it allows better assessment of the presence or absence of enhancing internal septae and mural nodules, and provides high quality internal signal intensity. The lesion’s local extent and nonovarian origin is also better visualized on MRI. 18 Although cross-sectional imaging is very helpful in providing preoperative assessment, most retroperitoneal lesions are defined during surgery. Macroscopically, the cyst wall could be smooth, stratified, thin or thick, or fibrous, depending on their origin. Additionally, the fluid could be clear, mucinous, or milky, depending on the etiology.

PRSC should be differentiated from other forms of cystic retroperitoneal lesions based on history and microscopic appearance, as well as its malignant form cystadenocarcinoma ( Table 1 ). Lee at al reviewed 56 cases of primary cystic mucinous neoplasms and reported that the presence of solid nodules in the cyst was the only statistically significant predictive factor of malignancy (cystadenocarcinoma). 19

Diagnostic fluid aspiration is discouraged due to concerns of seeding during the procedure if the lesion is malignant. The treatment of choice is complete surgical excision. The type of surgical intervention depends on the location, size, and expertise of the surgeon. Historically, laparotomy and complete cyst enucleation has been the recommended treatment approach. However, laparoscopic cyst excision is becoming more popular in recent years. Marsupialization and partial excision of the cysts or fluid drainage are avoided given higher risks for recurrence as well as the need for repeat surgical procedures. Obscure walls and location in close proximity of major blood vessels and organs are the main challenges during RPC excision. Recurrence can occur in approximately 25% of cases of RPCs according to a case series. 20 In our case, we have used laparoscopic approach with complete cyst excision. In cases of cystadenocarcinoma originating from the female reproductive organs, several case reports suggest empiric total abdominal hysterectomy and bilateral salpingo-oophorectomy with or without adjuvant chemotherapy. 21 22

## Conclusion

PRSCs are extremely rare lesions and oftentimes remain asymptomatic until they attain a very large size. Although advances in CT and MRI techniques enable us to identify various cystic lesions of the retroperitoneum, the exact diagnosis is based on histology and requires high clinical suspicion as well as expertise. We believe that endoscopic ultrasound may have a promising role in diagnosis, although further large prospective studies are required to confirm that. All attempts should be made to completely remove the cysts as risk of recurrence is high in partially excised lesions. Malignant lesions should be treated more aggressively with radical surgery and chemotherapy. More research is needed to better understand the pathogenesis of this entity.
